# Identifying and overcoming challenges in the EMA’s qualification of novel methodologies: a two-year review

**DOI:** 10.3389/fphar.2024.1470908

**Published:** 2024-11-14

**Authors:** Ana Drmić, Riccardo Saccà, Thorsten Vetter, Falk Ehmann

**Affiliations:** ^1^ Independent Researcher, Strasbourg, France; ^2^ Faculty of Health, Medicine and Life Sciences (FHLM), Maastricht, Netherlands; ^3^ European Medicines Agency, Amsterdam, Netherlands

**Keywords:** qualification of novel methodologies, regulatory science, european medicines agency, context of use, biomarker qualification

## Abstract

The EMA Qualification of Novel Methodologies procedure qualifies methods, technologies and methodologies within a well-defined context of use in a pharma R&D context based on the evaluation of the presented scientific rationale and submitted data. This policy brief analyses QoNM submissions providing policy messages and recommendations to stakeholders on how to better prepare qualification applications in this regard. The recommendations include: 1. Grounding validation strategy using a current standard measure or a distribution technique. 2. Accurately represent pertinent subgroups via accurate inclusion and exclusion criteria. 3. Establish a well-defined and specific CoU with clear descriptions of the use within a development program target population and disease stage. Lastly, it emphasizes role of the QoNM procedure in advancing medicine development methodologies within the EU.

## 1 Introduction

The EMA serves as the central regulatory authority for medicines in the EU, overseeing the benefit/risk assessment for authorization and monitoring of medicines (A pharmaceutical strategy for Europe, 2024; European Medicines Agency, 2020). EMA also supports the development of medicines through various mechanisms, such as support for early access, scientific advice and protocol assistance and pediatric procedures. One of these mechanisms is the Qualification of Novel Methodologies (QoNM) procedure. This is provided by the EMA’s Committee for Medicinal Products (MPs) for Human Use (CHMP) based on recommendations from its Scientific Advice Working Party (SAWP). The review and assessment of requests is performed by a Qualification Team (QT), a group of experts appointed based on the specific expertise requirements. The QT is usually led by two rapporteurs (CHMP and/or SAWP member) and reports to SAWP and CHMP (Committee for medicinal products for human use, 2024). There can be two outcomes depending on the appropriateness of the presented evidence to support Qualification.

• CHMP qualification opinion (QO), public document
• CHMP qualification advice (QA) on future protocols and studies to be performed for future qualification, confidential.

The QT drafts a List of Issues (LoI) which provides a preliminary scientific discussion and summarizes problems that have been identified in submitted qualification plans to be addressed by the applicant during a discussion meeting.

A LoI is the first readout of the assessment of a Qualification proposal that summarizes the scientific considerations and challenges that should be addressed by the applicant to achieve qualification or perform an optimized the qualification exercise going forward (in the context of a QA).

LoI include questions or concerns related to the validation strategy, reliability, accuracy and reproducibility of the methodology, as well as regulatory considerations that may need to be addressed. With them, applicants are provided with a focused overview of the limitations and considerations related to the specific qualification development, which combined with QA letters, can be a useful guide for the researchers to improve their qualification application and to have their methodology qualified.

To ensure transparency, draft QOs are shared before the final opinion is published (Table 1). Complementing a confidential QA letter, EMA may propose publishing a letter of support when a novel cannot yet be qualified based on the submitted data (Qualification of novel methodologies for drug development: guidance to applicants, 2024). These letters aim to encourage data-sharing and collaboration, enabling studies for the methodology’s eventual qualification and can also be leveraged for fundraising (Qualification of novel methodologies forb).

**TABLE 1:** Published qualification opinions given by EMA’s CHMP based on recommendations by the SAWP. Usually most of the Qualification advice are confidential and in accordance with EMA policy are not made publicly available. This table lists the few exceptions that have been made public and are available on the EMA website due to the desires of the applicants.

Published QOs	First published
Qualification opinion for Centiloid measure of Amyloid PET to quantify brain amyloid deposition	25/06/2024
GFR slope as a Validated Surrogate Endpoint for RCT in CKD	21/12/2023
Stride velocity 95th centile as primary endpoint in studies in ambulatory Duchenne Muscular Dystrophy	31/07/2023
iBox Scoring System as a secondary efficacy endpoint in clinical trials investigating novel immunosuppressive medicines in kidney transplant patients	16/12/2022
Use of Enroll-HD (a Huntington’s disease patient registry) as a data source and infrastructure support for post-authorisation monitoring of medical products	28/07/2022
Prognostic Covariate Adjustment (PROCOVA™)	20/09/2022
Islet Autoantibodies (AAs) as Enrichment Biomarkers for Type 1 Diabetes (T1D) Prevention Clinical Trials	31/03/2022
IMI PREFER	03/05/2022
Multiple sclerosis clinical outcome assessment (MSCOA)	02/03/2020
Treatment effect measures when using recurrent event endpoints	14/04/2020
eSource Direct Data Capture (DDC)	19/09/2019
Stride velocity 95th centile as a secondary endpoint in Duchenne Muscular Dystrophy measured by a valid and suitable wearable device	29/05/2019
Cellular therapy module of the European Society for Blood and Marrow Transplantation (EBMT) Registry	28/02/2019
The European Cystic Fibrosis Society Patient Registry (ECFSPR) and CF Pharmaco-epidemiology Studies	03/10/2018
Molecular neuroimaging of the dopamine transporter as biomarker to identify patients with early manifest Parkinsonism in Parkinson’s disease	19/07/2018
Plasma fibrinogen as a prognostic biomarker (drug development tool) for all-cause mortality and COPD exacerbations in COPD subjects	02/05/2018
Proactive in COPD	19/04/2018
Paediatric ulcerative colitis activity index (PUCAI)	20/01/2016
Ingestible sensor system for medication adherence as biomarker for measuring patient adherence to medication in clinical trials	15/02/2016
Total kidney volume (TKV) as a prognostic biomarker for use in clinical trials evaluating patients with autosomal dominant polycystic kidney disease (ADPKD)	13/11/2015
Exacerbations of chronic pulmonary disease tool (EXACT), and EXACT-respiratory symptoms measure (E-RS) for evaluating treatment outcomes in clinical trials in COPD	13/04/2015
*In-vitro* hollow fiber system model of tuberculosis (HFS-TB)	06/02/2015
MCP-Mod as an efficient statistical methodology for model-based design and analysis of phase-II dose-finding studies under model uncertainty	10/02/2014
A novel data-driven model of disease progression and trial evaluation in mild and moderate Alzheimer’s disease	03/10/2013
Alzheimer’s disease novel methodologies/biomarkers for the use of cerebrospinal-fluid amyloid beta 1–42 and t-tau and/or positron-emission-tomography amyloid imaging (positive/negative) as biomarkers for enrichment	04/04/2012
Low hippocampal volume (atrophy) by magnetic-resonance imaging for use in clinical trials for regulatory purpose in predementia stage of Alzheimer’s disease	09/12/2011
Novel methodologies in the predementia stage of Alzheimer’s disease: cerebrospinal-fluid-related biomarkers for drugs affecting amyloid burden	16/05/2011
Alzheimer’s disease novel methodologies/biomarkers for BMS-708163	10/02/2011
ILSI/HESI submission of novel renal biomarkers for toxicity	26/11/2010
Final conclusions on the pilot joint European Medicines Agency/Food and Drug Administration VXDS experience on qualification of nephrotoxicity biomarkers	22/01/2009

Successful QoNM supports and enables the development of novel medicines. The qualification process ensures that methodologies meet the necessary standards, e.g., validity, reliability, specificity and precision, required for drug development and regulatory decision making. QONM establishes the reliability and validity of these tools and helps integration and alignment of the qualified innovative approaches with the regulator so they can be considered by the CHMP during regulatory submissions.

The QoNM promotes accessibility within the scientific community. Through the publication of QOs, stakeholders gain insights into approved methodologies, their context of use (CoU) in the development of MPs, and the evidence supporting qualification.

The CoU refers to the specific intended use of a novel methodology in the development of MPs, including, e.g., the target population, the specific disease or condition, and the specific stage of development in which the methodology will be used. It is an important consideration in the QoNM, as a clear and concise description of the intended use is critical for regulatory assessment of the supportive evidence. This helps to ensure that the novel methodology is being used appropriately and effectively, and that it meets the necessary regulatory requirements.

CHMP, based on recommendations by SAWP, has used this procedure to assess regulatory acceptability for innovative methods, such as biomarkers, imaging methods, clinical outcome assessments, new animal models, statistical methods, innovative trial methodologies and big data approaches. However, the qualification of these methods is often compromised by weaknesses of applications and limitations tackled in this study. The subject of the LoI search are submissions that had an initial CoU for the proposed novel methodology, later revised by the CHMP, and that have been accepted for a QO, but have not necessarily been successful, which are identified as “agreeable CoU” in the report.

## 2 Methodology

To identify the problematic aspects of qualification of applications, a review of regulatory documents, scientific literature, and guidance from the CHMP was conducted (Essential considerations for successful qualification, 2017). The views expressed in this article are the personal views of the authors and may not be understood or quoted as being made on behalf of or reflecting the position of the regulatory agency/agencies or organisations with which the authors are employed/affiliated. A major section of the items reviewed covers the LoI processed in the period from 1 January 2021 until the 20th of December 2022.

These documents provide a preliminary scientific discussion as well as raising issues to prepare a discussion meeting between QT and the applicant, of all 43 assessed procedures were extracted from EMA’s internal database.

Each LoI was examined, and the weaknesses of each qualification were split into major categories that appeared to be the most frequent among all applications. The review focused on the most common methodologies used in the development of MPs, such as biomarkers (Kraus, 2018; Gromova et al., 2020), imaging methods (Vermeulen et al., 2022; Izmailova et al., 2023) clinical outcome assessments (Clinical Outcome Assessments | U, 2021; Clinical outcomeassessment, 2021), statistical methods (Peterson and Altan, 2016; Kuhn et al., 2016), innovative trial methodologies (Innovative Science and Technology Approaches; Orloff et al., 2009; Beckman et al., 2022) and big data approaches (Qian et al., 2019; Zhu, 2020). Problematic aspects arising during the evaluation of novel qualifications include the validation strategy, target population and CoU as the most common ones. The summary of recommendations on how to address them are shown in Table 2.

**TABLE 2:** Observed arising issues in the QoNM applications with recommendations for addressing them. After discussing with CHMP and SAWP experts in the procedure, it was decided to not focus of functional endpoints and clinical trial application and conduct as, despite their incidence, they were considered relatively niche issues, something that would have probably been more apparent if a larger timeframe was analysed.

Issue	Recommendation
Validation strategy	- anchoring the method to a current standard measure or using distribution-based techniques - consider whether to use learn and confirm in separate datasets or cross-validation in pre-specified sections of one overall database
Target population	- essential to avoid inappropriate inclusion/exclusion criteria, poor representation of relevant subgroups (e.g., age, gender, ethnicity), and inadequate consideration of comorbidities or other relevant factors that may affect treatment outcomes
Context of use	- crucial to have sufficient evidence supporting the intended CoU and scope of the method, along with a clear definition or description of the target population, type of measure, phase of development in which measure will be applied or disease condition

A document containing a list of QoNM procedure applications processed in the interest interval was downloaded from EMA’s internal database. At the time of the analysis all the authors were affiliated with the Agency. Procedures that never started were excluded from the analysis. 43 procedures were examined, each being assessed individually on their intended and agreeable CoU, as well as a list of problematic aspects of qualification applications and a search for applications in clinical trials (CTs), related publications, or registration of patents to identify the research, development and impact activities surrounding the respective CoUs.

After identifying the applications on the EMA internal database, IRIS, within the desired timeframe, the document produced by both applicants, SAWP and CHMP were examined and a content analysis was performed. The topics discussed were identified and categorized and the intended CoU proposed by the applicant were compared to the agreeable CoU presented after modifications were required from CHMP.

Under the assumption that each qualification procedure is likely related to existing patents, publications and/or CTs, we performed searches in all three categories using the same specific set of keywords related to the methodology that has been qualified as part of a successful QO.

CT searches were executed using the ClinicalTrials.gov database, utilizing the expert search function.

Publication searches were conducted in the PubMed database, while patent searches were carried out in the Espacenet database, with filters employed to narrow the search to title, abstracts and claims as including other documents such as description resulted in too many irrelevant hits.

## 3 Results

This data analysis led to three main findings regarding CoU, LoIs and the research, development and impact activities surrounding them, demonstrated through search results for publications, patents and CTs. The identified lists of issues were categorized, with validation strategy, doubts concerning the link between measurements and the predictive variables, being the most frequently identified issue category, appearing in nearly 50% of the procedures. Other commonly occurring problematic aspects of qualification applications include.

• Target population
• The inability to have a relevant and representative study population/subgroup.
• Context of use
• Major issues concerning the characterization of the CoU presented by the applicant.
• Functional endpoint(s)
• Issues concerning the endorsement of a novel endpoint in the context of EMA’s CMA.
• CT application and conduct
• Issues concerning the application of the trial protocol and the resulting lack of data integrity, proper documentation, and traceability of results.

**FIGURE 1:**
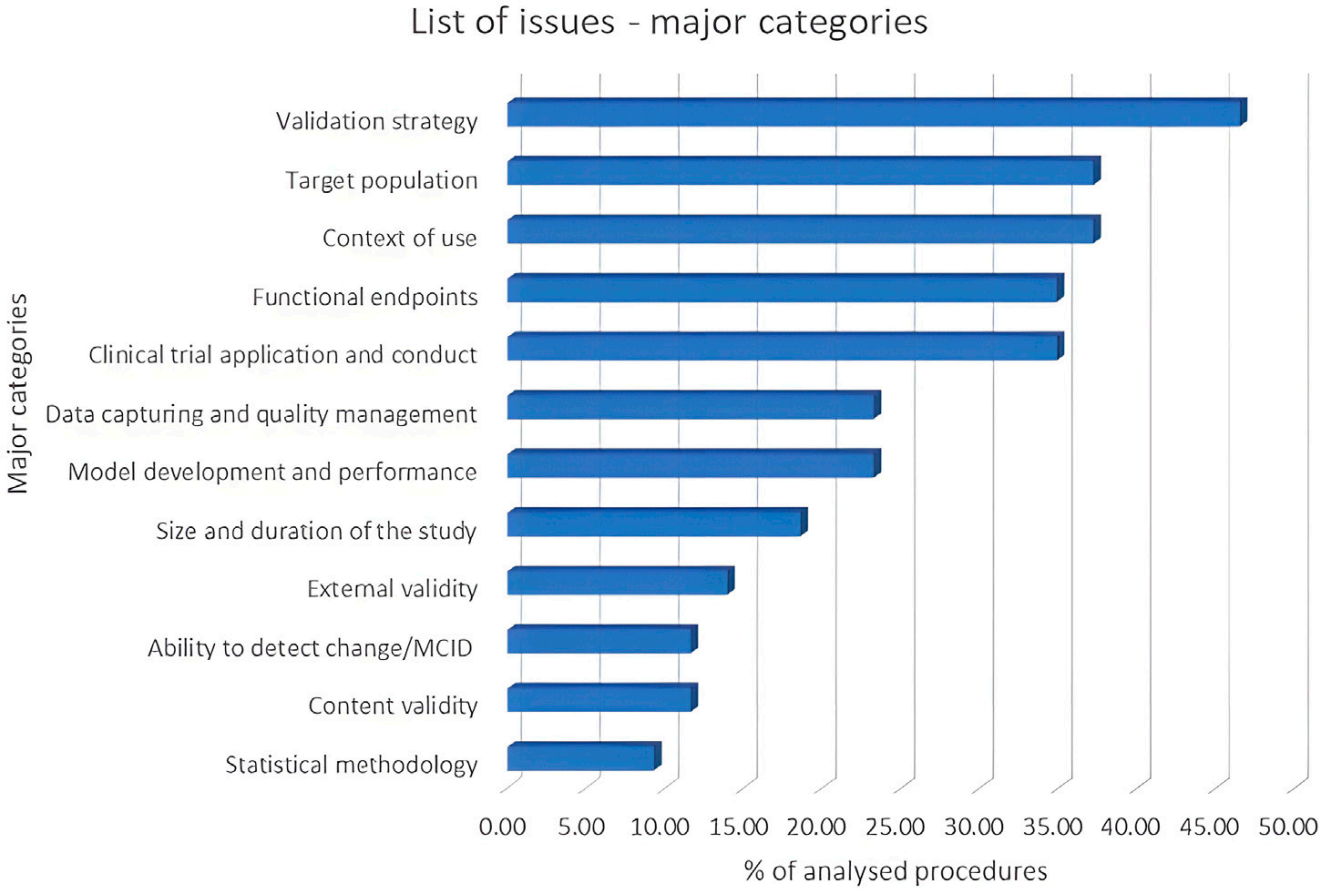
Problematic aspects encountered in the qualification applications–major weaknesses. Analysing the 43 QoNM procedures between 2021–2022 has resulted in the identification of several recurring issue categories mentioned by the applicants, the five most prevalent of which appear in more than 30% of the total procedures.

Additionally, there are also other weaknesses, that are listed in Figure 1. While all these five weaknesses appear in more than 30% of the applications examined, after discussing with CHMP and SAWP experts in the QoNM procedure, it was decided to not focus of functional endpoints and CT application and conduct as, despite their incidence, they were considered a relatively niche issues, something that would have likely been clearer if a larger timeframe was analyzed.

The study identified four categories of QoNM submissions according to the CoU: New clinical outcome assessment, new digital tools and imaging methodologies, new biomarker qualification and new statistical methodologies. Digital Tools and imaging methodologies were grouped together due to their observed overlap and similarity in multiple applications where the QA/QO primarily focused on the digital component of the methods, technologies and methodologies. New clinical outcome assessments were found to be the most researched category, covering 13 out of 43 submissions each.

**FIGURE 2:**
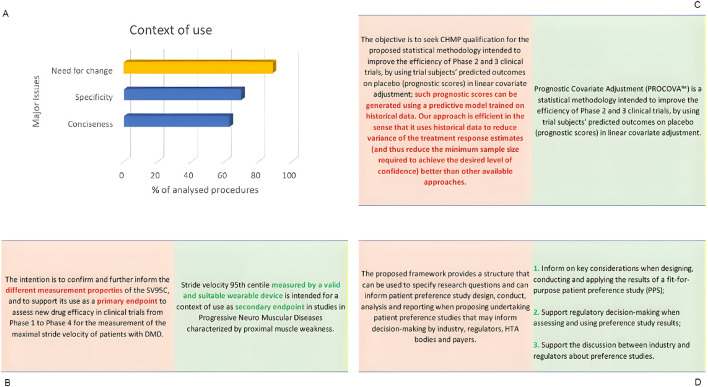
Problematic aspects encountered in the qualification applications–weaknesses of the proposed CoU. Another aspect of the QoNM applications analysis focused on the various CoU presented to the CHMP, what they were lacking and how they were improved to become “agreeable”. The figure presents **(A)** the distribution of the most prevalent issues in the presented CoU **(B–D)** Example of modified context of use–comparison between proposed (red) and agreed (green) versions.

When it comes to comparing the proposed and agreeable CoU, analysis showed that in nearly 90% of these 43 procedures CHMP required a certain change in the proposed CoU, particularly regarding the lack of conciseness and specificity, as seen in the examples of Figures 2A–D.

Following the initial drug development, the initial findings are usually published. Further evidence generation (e.g., CTs) is conducted at later stages of development. Patents play a crucial role in securing innovation and later marketplace, as well as attracting funding opportunities.

To reach a point of applying for a QoNM, extensive research already had to be done considering protecting intellectual property, publishing research results and performing CTs. That is why it was decided to perform a search of each category to try to describe the research, development and impact activities surrounding the respective CoU.

Searching for publications, patents, and CTs related to agreeable CoU qualified for a QO in QoNM provides a comprehensive understanding of the scientific and regulatory landscape surrounding the methodology. This can help identify challenges or limitations of the qualification process and aligns with the EMA’s objective of enabling and leveraging research and innovation in regulatory science. Additionally, can facilitate the identification of gaps in knowledge, leading to a more thorough understanding of the methodology and its applications.

The search results, shown in Table 3, represent the number of search hits for patents, publications and CTs for each CoU. Search terms derived from CoU. Several cases, the ones with a QO, were investigated further to determine if they were applied in line with the agreeable CoU, as they are already in the public domain and do not raise confidentiality issues.

**TABLE 3:** Submissions split into categories according to CoU with the number of hits for each search of patents, clinical trials and publications. CoU presented in a form shortened and anonymized by authors. Original CoU for procedures with a QO is published together with QO on EMA’s official website. Documents of QA are confidential. The numbers in the table are referring to the number of hits resulting from each search. CT–clinical trials, PubMed–publications retrieved from PubMed database.

Context of use	Patents	CT	PubMed
New Clinical Outcome Assessments			
Novel clinical endpoints for use in intermediate age-related macular degeneration (iAMD) based on anatomical changes	0	1	11
Novel clinical endpoints in patients with iAMD examining measurement properties, and correlations between consolidated endpoints and patient-reported health	5	4	7
Novel endpoints of Atopic Dermatitis measuring night-time scratch and sleep using wearable technology	0	1	0
Using Psoriatic Arthritis Disease (PASD) Activity Score and Minimal Disease Activity Score (MDA) as assessment tool for PASD.	11	19	22
Utilizing a disease specific patient reported outcome as a tool to assess change in symptoms and impacts over time in patients with pulmonary arterial hypertension	0	19	12
Utilize a scoring system 1-year post-transplant as a surrogate endpoint for the 5-year risk of death-censored allograft loss in kidney transplant subjects	1	0	1
PBPK based methodology to replace therapeutic equivalence studies for CNS drugs	0	0	1
Internationally harmonized score to support acceptability of oral/buccal medicines in children >12	0	0	1
Clinical trial simulation platform to optimize clinical studies in Duchenne’s Muscular Dystrophy	0	0	1
Master protocol for a platform trial to determine the safety and efficacy of immunotherapeutic IMPs targeting the preservation of β cell function in newly diagnosed people with T1DM	1	0	1
Master protocol for the evaluation of investigational medicinal products in high-risk family members of patients with T1D	0	3	35
Tool to assess treatment efficacy of drugs for primary Sjögren’s syndrome (pSS) based on improvement of disease activity	0	2	1
Novel efficacy endpoints and PROs to assess treatment benefit in drug clinical trials for the treatment of Achromatopsia (ACHM)	6	2	60
	24	51	153
New Digital Tools and Imaging Methodologies			
Wearable derived endpoints for chronic heart failure (CHF)	0	7	7
Stride velocity 95th centile measured by a wearable device as a secondary endpoint in Progressive Neuro Muscular Diseases	0	5	5
Digitally derived clinical outcome assessment measuring nocturnal scratch as a secondary or exploratory endpoint in clinical trials of moderate and severe Atopic Dermatitis	0	1	0
Deep learning algorithm applied to histological slides of mesothelioma and HCC to derive covariates to optimise efficacy analysis in RCTs	1	1	4
Machine learning based automated clinical event adjudication in clinical trials	0	0	2
Novel app-based assessment tool/digital biomarker to assess disease domains of Patients with Multiple Sclerosis	3	0	2
Outcome measures from a monitoring application to remotely measure the motor and cognitive signs and symptoms in Huntington’s disease patients to support the development of novel products for the treatment of HD.	0	0	0
Electronic daily diary for patients with sickle cell disease to self-report vaso-occlusive crisis	0	27	538
Model-based CT Simulation Platform to Optimize Design of Efficacy Evaluation Studies in Parkinson’s Disease	0	0	3
Web platform based on calibrated and standardised non-invasive Diffusion Tensor magnetic resonance Imaging (DTI) measurements of cerebral white matter intended to study white matter in neurological or neurodegenerative diseases linked to white matter alterations	0	0	336
Patient-specific model that predicts the absolute risk of fracture of the proximal femur in case of fall	0	0	4
Digital biomarkers to measure efficacy of treatment of fatigue, sleep quality and impact of sleep disturbances in patients with neurodegenerative disorders (NDD) and immune-mediated inflammatory diseases (IMID)	0	0	1
	4	41	902
New Biomarker Qualification			
Machine-Learning based diagnostic biomarker to assess the Non-alcoholic Fatty Liver disease activity score (NAS) components and fibrosis stage in liver biopsies in non-alcoholic steatohepatitis (NASH) clinical trials	1	1	8
Novel biomarker to monitor disease activity possibly related to axonal damage and assess treatment responses in paediatric neurological diseases	0	0	3
Prognostic or pharmacodynamic/response biomarker in progressive multiple sclerosis Clinical Trials	3	0	4
Novel biomarker or a composite panel of biomarkers that aids in identifying subjects with potential acute liver injury caused by drugs	0	1	10
Tool to quantify Cystic Fibrosis Transmembrane Conductance Regulator (CFTR) protein activity in patients with Cystic Fibrosis using rectal organoids	0	0	4
Novel biomarker, or biomarker panel, to aid in the detection of acute exocrine pancreas injury in phase I trials of drugs that may induce pancreas injury (DIPI)	0	0	2
	4	2	31
New Statistical Methodologies			
Performing post authorisation safety studies (PASS) based on secondary use of info from a data network consisting of six participating multiple sclerosis registries	0	0	2
Data source and infrastructure for Enroll-HD registry-based studies for Huntington’s disease	5	8	110
Self-reported questionnaire to assess anhedonia in subjects with major depressive disorder to evaluate treatment benefits	0	5	11
Risk and response score in diabetic patients with chronic kidney disease	21	11	187
Tool to measure best corrected visual acuity (BCVA) in future CTs	0	6	463
Methodology to collect real-world data and create a Spinal muscular atrophy (SMA) core dataset to support research and regulatory decision-making	0	0	2
Statistical methodology using prognostic scores intended to improve the efficiency of clinical trials	0	0	3
Self-reported questionnaires use as efficacy endpoint(s) in Crohn’s disease (CD) and ulcerative colitis (UC) drug development trials	0	1	1
Framework to specify research questions and inform patient preference studies	2	1	14
Clinical platform trial protocol to evaluate the safety and efficacy of novel agents in combination with existing therapies for the treatment of young patients with relapsed or refractory B-cell Non-Hodgkin Lymphoma (B-NHL)	0	2	29
Patient and observer reported outcome measure to capture symptoms of paediatric patients with Ulcerative Colitis in clinical studies	0	0	3459
	28	34	4281

## 4 Discussion

In 2022 Bakker et al. (Bakker et al., 2022) published a study examining the EMA’s biomarker qualification procedures from 2008 to 2020, investigating frequency, outcomes, challenges and biomarker characteristics. They emphasize the importance of robust validation strategies in discussions between applicants and regulators. Another study by Hendrikse et al. (Hendrikse et al., 2022) also examined biomarkers in the EU regulatory system in the same period, focusing on interactions between applicants and the EMA.

The research and the analysis results highlight the need for increased EMA support and showcases the importance of regulatory qualification for precision medicine and patient benefits. As seen from the number of submissions, new biomarker qualifications are still relevant. However, other categories of methodologies have also emerged, and the previously mentioned studies could serve as examples of a more thorough investigation that could be performed for each category in the future.

QoNM is a clearly defined, yet complex, process that requires planning and collaboration. The findings of this study, presented at the EMA multi-stakeholder workshop on QoNM (EMA multi, 2023) in April 2023, highlight the importance of the CoU, the challenges faced during qualification, namely, an inadequately described and justified CoU, validation strategy, target population, and the research, development and impact activities surrounding the respective CoU. By aligning with the EMA’s strategic plan, the qualification process can support the integration of science and technology, foster collaborative evidence generation, and enhance regulatory science within the EU.

Analysis of the identified CoU statements in QoNM submissions reveals a strong interest of applicants in clinical outcome assessment, statistical methodologies, and innovative digital and imaging tools together with new biomarkers and ATMP. Observing the difference between proposed and agreeable suggest that clear, specific and concise definition of the CoU is crucial in ensuring accurate and appropriate use of novel methodologies.

One example of a non-specific CoU proposed by the applicant (Stride velocity 95th centile as a secondary endpoint in Duchenne Muscular Dystrophy (DMD) measured by a valid and suitable wearable device, QO published (Opinions and letters of support on the qualification of novel methodologies for medicine development, 2024)) stated that the goal was to validate and enhance the measurement properties of SC95C and establish it as the primary endpoint in CTs across all phases, aimed at assessing the efficacy of new drugs for DMD patients by measuring maximal stride velocity. As the evidence did not match the targeted claim, a modification of the CoU was necessary, from primary to secondary endpoint (Figure 2B). Also, afterwards, the applicant requested to expand the CoU of SV95c to a secondary (efficacy) endpoint for other progressive neuromuscular diseases characterized by proximal muscle weakness. Nonetheless, the evidence presented was not supportive enough to validate SV95c as a secondary endpoint across different NMDs, as a result It was concluded that only the use of SV95C as an exploratory endpoint in NMD CTs could be supported. This highlights that clear and specific communication is crucial in ensuring developers and regulators reach a common understanding and that the intended use as supported by the presented evidence is accurately represented. Although not part of the analysis the time frame, should be mentioned that in July 2023 a new QO (European Medicines Agency, 2024) was published where SV95cCoU is extended to primary efficacy endpoint in DMD CTs, which indicates the value of a successful, stepwise qualification exercise for the applicant.

Another example, Prognostic Covariate Adjustment (PROCOVA™, QO published (Opinions and letters of support)), highlights the importance of focusing on the main points and not going beyond necessary. The applicant, in this case, overexplains why the proposed methodology is optimal, expanding the proposed CoU with no additional relevant information for this stage (Figure 2C).

The third example (IMI PREFER) highlights the need to specify the intended use in a concise way. The proposed CoU is more complex and lists several points in one long sentence. In comparison, the qualified CoU is more straightforward and split into bullet points to allow easier comprehension of the main themes (as seen from the QO (Opinions and letters of support)) (Figure 2D).

Since a specific set of keywords was chosen for the search, based on the agreed CoU for each submission, it helped narrow down the scope. Nonetheless, it needs to be considered the possibility that not all the hits in the presented Table 3 correspond to the methodology in question, on the contrary, the higher the number of hits, especially when in PubMed, of the higher is the possibility of finding false positives, despite the keyword set matching.

For most submissions, there was a greater number of hits for publications in comparison to the ones for CTs and patents. Although we are talking about new methodologies, certain information has already been published before and after the qualification date, resulting in more hits on PubMed. CTs showed few hits and even fewer related to the novel methodology in question. For example, if we look at data source and infrastructure for Enroll-HD registry-based studies for Huntington’s disease (Table 3) we see eight hits for CTs. However, exploring further, we discovered that only two were related to this novel methodology and its CoU, and one was in the recruiting stage at the time of this analysis.

Looking into the second example, utilize a scoring system 1-year post-transplant as a surrogate endpoint for the 5-year risk of death-censored allograft loss in kidney transplant subjects. (Table 3). Although no relevant CTs were found, a publication was identified describing an observational cohort study in line with the CoU. The fewer numbers can be explained by considering that the analysis only covered the previous 2 years and that these methodologies are new and innovative, therefore existing published data might be limited, and potential CTs might happen in the future. Patent search gave the least number of hits in total, which might also be explained by the timeframe. Another possible explanation could also be that a more refined search is necessary to get reliable information. It should be kept in mind that, while this analysis gives a rough overview of the journey of each novel methodology before the qualification stage, it is hampered by its scope and time limitations. As a result, the topic could be investigated further, and a deeper and more individually tailored analysis over a longer time would benefit the findings.

## 5 Conclusion

The analysis of 43 QoNM procedures between 2021–2022 highlighted several important findings and recommendations.

QoNM can be divided four different categories based on the agreeable CoU. Several weaknesses were identified, the most prevalent being to validation strategy, target population and CoU, all appearing in more than 35% of the applications.

The analysis highlighted concerns over validation strategy, appropriate selection of target population, relevant factors affecting treatment outcomes, justification of intended use and scope in QoNM procedures that applicants should carefully consider before the assessment. The analysis of the LoIs shall guide future applicants to optimize their submission package enabling an efficient Qualification procedure.

Aside from looking at these applications, the research, development and impact activities surrounding these CoU were also discussed, emphasizing the importance of monitoring publications, patents, and CTs to observe if the novel methodologies were employed in line with the agreeable CoU.

While the scope and time limitations of the analysis impede the development of a comprehensive understanding of the scientific and regulatory landscape surrounding QoNM, we believe that potential follow-up studies could be promising. Expanding the scope and timeframe of the analysis could enable the identification of additional challenges, limitations and supports the development of cases for regulatory approval and aid in identifying areas for further development, enhancing the overall understanding of the methodology and its potential applications. In summary, this analysis provides some high-level insights into the processes of Qualification of Novel Methodologies and offers recommendations to improve the content of QoNM applications and, consequently, higher likelihood of successful qualification. It is of value for researchers and regulatory authorities to consider these findings and recommendations to improve the qualification process and advance.

## References

[B17] A pharmaceutical strategy for Europe - European Commission. 2024 Available at: https://health.ec.europa.eu/medicinal-products/pharmaceutical-strategy-europe_en.

[B1] Bakker E. Hendrikse N. M. Ehmann F. van der Meer D. S. Llinares Garcia J. Vetter T. (2022). Biomarker qualification at the European medicines agency: a review of biomarker qualification procedures from 2008 to 2020. Clin Pharmacol Ther 112 (1), 69–80. 10.1002/cpt.2554 35137949 PMC9313861

[B2] Beckman R. A. Natanegara F. Singh P. Cooner F. Antonijevic Z. Liu Y. (2022). Advancing innovative clinical trials to efficiently deliver medicines to patients. Nat Rev Drug Discov. 21 (8), 543–544. 10.1038/d41573-022-00109-y 35760887 PMC9834420

[B3] Clinical outcome assessments | U.S. Department of Health and Human Services; 2021 Available at: https://toolkit.ncats.nih.gov/module/prepare-for-clinical-trials/working-with-industry-to-design-clinical-trials/clinical-outcome-assessments/#:∼:text=Clinical%20outcome%20assessments%20%28COAs%29.

[B4] Clinical outcomeassessment (coa) compendium. Center for drug evaluation and research/FDA; (2021). Available at: https://www.fda.gov/drugs/development-resources/clinical-outcome-assessment-compendium.

[B5] Committee for medicinal products for human use (CHMP) (2024). European Medicines Agency. Available at: https://www.ema.europa.eu/en/committees/committee-medicinal-products-human-use-chmp.

[B6] EMA multi-stakeholder workshop on qualification of novel methodologies | European Medicines Agency (2023). Available at: https://www.ema.europa.eu/en/events/ema-multi-stakeholder-workshop-qualification-novel-methodologies.

[B7] Essential considerations for successful qualification of novel methodologies | European Medicines Agency. 2017. Available at: https://www.ema.europa.eu/en/documents/other/essential-considerations-successful-qualification-novel-methodologies_en.pdf.

[B20] European Medicines Agency (2024). Available at: https://www.ema.europa.eu/en/qualification-novel-methodologies-medicine-development.

[B22] European Medicines Agency (2020). Available at: https://www.ema.europa.eu/en/about-us/what-we-do#facilitate-development-and-access-to-medicines-12354.

[B8] Gromova M. Vaggelas A. Dallmann G. Seimetz D. (2020). Biomarkers: opportunities and challenges for drug development in the current regulatory landscape. Biomark Insights 15, 1177271920974652. 10.1177/1177271920974652 33343195 PMC7727038

[B9] Hendrikse N. M. Llinares Garcia J. Vetter T. Humphreys A. J. Ehmann F. (2022). Biomarkers in medicines development—from discovery to regulatory qualification and beyond. Front Med 9, 878942. 10.3389/fmed.2022.878942 PMC9086587 35559349

[B10] Innovative science and technology approaches for new drugs (ISTAND) Pilot program | FDA. Available at: https://www.fda.gov/drugs/drug-development-tool-ddt-qualification-programs/innovative-science-and-technology-approaches-new-drugs-istand-pilot-program.

[B11] Izmailova E. S. Maguire R. P. McCarthy T. J. Müller MLTM Murphy P. Stephenson D. (2023). Empowering drug development: leveraging insights from imaging technologies to enable the advancement of digital health technologies. Clin Transl Sci 16 (3), 383–397. 10.1111/cts.13461 36382716 PMC10014695

[B12] Kraus V. B. (2018). Biomarkers as drug development tools: discovery, validation, qualification and use. Nat Rev Rheumatol 14 (6), 354–362. 10.1038/s41584-018-0005-9 29760435

[B13] Kuhn M. Yates P. Hyde C. (2016). Statistical methods for drug discovery. Nonclinical Statistics Pharm. Biotechnol. Industries. 53–81. 10.1007/978-3-319-23558-5_4

[B14] Opinions and letters of support on the qualification of novel methodologies for medicine development (2024). European Medicines Agency Available at: https://www.ema.europa.eu/en/human-regulatory-overview/research-development/scientific-advice-protocol-assistance/opinions-letters-support-qualification-novel-methodologies-medicine-development.

[B15] Orloff J. Douglas F. Pinheiro J. Levinson S. Branson M. Chaturvedi P. (2009). The future of drug development: advancing clinical trial design. Nat Rev Drug Discov 8 (12), 949–957. 10.1038/nrd3025 19816458

[B16] Peterson J. Altan S. (2016). Overview of drug development and statistical tools for manufacturing and Testing, 383–414.

[B18] Qian T. Zhu S. Hoshida Y. (2019). Use of big data in drug development for precision medicine: an update. Expert Rev Precis Med Drug Dev. 4 (3), 189–200. 10.1080/23808993.2019.1617632 31286058 PMC6613936

[B19] Qualification of novel methodologies for drug development: guidance to applicants (2024). Available at: https://www.ema.europa.eu/en/documents/regulatory-procedural-guideline/qualification-novel-methodologies-drug-development-guidance-applicants_en.pdf.

[B21] Vermeulen I. Isin E. M. Barton P. Cillero-Pastor B. Heeren R. M. A. (2022). Multimodal molecular imaging in drug discovery and development. Drug Discov Today 27 (8), 2086–2099. 10.1016/j.drudis.2022.04.009 35429672

[B23] Zhu H. (2020). Big data and Artificial Intelligence modeling for drug discovery. Annu. Rev Pharmacol Toxicol 60, 573–589. 10.1146/annurev-pharmtox-010919-023324 31518513 PMC7010403

